# Mid-arm muscle circumference: A significant factor of all-cause and cancer mortalities in individuals with elevated platelet-to-lymphocyte ratio

**DOI:** 10.1371/journal.pone.0208750

**Published:** 2018-12-13

**Authors:** Pi-Kai Chang, Wei-Liang Chen, Li-Wei Wu

**Affiliations:** 1 Division of Colon and Rectal Surgery, Department of Surgery, Tri-Service General Hospital, National Defense Medical Center, Taipei, Taiwan, Republic of China; 2 Graduate Institute of Medical Sciences, National Defense Medical Center, Taipei, Taiwan, Republic of China; 3 School of Medicine, National Defense Medical Center, Taipei, Taiwan, Republic of China; 4 Division of Geriatric Medicine, Department of Family and Community Medicine, Tri-Service General Hospital, National Defense Medical Center, Taipei, Taiwan, Republic of China; 5 Division of Family Medicine, Department of Family and Community Medicine, Tri-Service General Hospital, National Defense Medical Center, Taipei, Taiwan, Republic of China; McGill University, CANADA

## Abstract

Platelet-to-lymphocyte ratio (PLR) is an inflammatory maker, and high PLR is associated with mortality in several diseases. The predictors of mortality in individuals with high PLR is still lacking. Our aims were to assess if mid-arm muscle circumference (MAMC) can predict all-cause mortality, cancer mortality, and cardiovascular mortality in individuals with high PLR. Adult participants from the National Health and Nutrition Examination Survey III (1988–1994) were included. All participants were divided into low PLR and high PLR groups with the cut-off point being the median PLR level, and each group was evaluated for risk factors of mortality. MAMC was divided into tertiles and the general characteristics of the study population related to MAMC were evaluated. The study included 14,221 adults with 6,701 (47.1%) male and 7,520 (52.9%) female participants. The median PLR ratio was 122. Higher levels of systolic blood pressure, total triglycerides, total cholesterol, low-density lipoprotein, C-reactive protein, uric acid, and glucose, as well as a higher age, were associated with increased risk of mortality in both groups. After adjusting for all the covariates, in the higher PLR group, the highest MAMC tertile was significantly associated with lower hazard ratios for all-cause and cancer mortalities compared with the lowest MAMC tertile. However, this association was not observed in the low PLR group. The highest MAMC tertile showed protective effects from all-cause and cancer mortalities compared with the lowest MAMC tertile in individuals with PLR ≥ 122. In conclusion, the highest MAMC tertile was significantly associated with decreasing HRs for all-cause and cancer mortalities compared with the lowest MAMC tertile in individuals with elevated PLR.

## Introduction

The platelet-to-lymphocyte (PLR) ratio is a universally known indicator of systemic inflammation. Previous literature has demonstrated that higher PLR is associated with poor prognosis and higher mortality in those with malignancies, cardiovascular diseases, and end-stage renal disease [1–4]. Anthropometric parameters have been widely used to screen for, or monitor, a disease. Mid-arm muscle circumference (MAMC) is calculated as: mid-upper arm circumference (MAC) (cm) - 0.3142 x triceps skinfold (TS) thickness (mm). It is a simple, inexpensive, and universally applicable measurement. MAMC correlates strongly with more accurate dual-energy X-ray absorptiometry measures of lean mass, and can represent lean mass well [5]. Meanwhile, sufficient muscle mass may be positively related to functional performance and survival [6–7]. Despite the growing interest and research, information on the relationships between anthropometric parameters and inflammatory markers is lacking. Therefore, the primary objective of this study was to examine the associations between PLR and MAMC. Furthermore, the secondary objective was to evaluate how MAMC levels affect all-cause mortality, cancer mortality, and cardiovascular mortality in people with low and high PLR. The data was obtained from the National Health and Nutrition Examination Survey (NHANES) III, which is a large-scale, nationally representative, and population-based database of adults in USA.

## Materials and methods

### Data source and participants

The National Health and Nutrition Examination III (1988–1994) was a complex multi-stage stratified study managed by the National Center for Health Statistics (NCHS) of the Centers for Disease Control and Prevention (CDC) [8]. NHANES III was a program to evaluate the health and nutritional statuses with household interviews and physical examinations of non-institutionalized adults in USA. It was conducted in accordance with the guidelines of the Declaration of Helsinki. A subsample of participants aged 20–90 years was identified from it with a mean follow-up of 14.3 years. The survey design and procedures (questionnaires, examinations, and laboratory components) of NHANES III are publicly available online at http://www.cdc.gov/nchs/nhanes.htm.

### Follow-up data on all-cause mortality, cancer mortality, and cardiovascular mortality

The mortality data of the NHANES III study is from a probabilistic match between NHANES III and the National Death Index (NDI) records. NDI is available publically and contains de-identified death-certificate data, updated through 31 December 2006.

The cause of death was identified according to the guidelines of the International Statistical Classification of Disease, Injuries and Causes of Death; the 9th revision was used for those who died before 1999 and 10th revision for all others. NHANES procedures harmonized the differences in definitions and causes of death, all of which have been demonstrated to be comparable.

We included deaths from all causes; we included deaths due to malignant neoplasms, which were coded from C00–C97 in the International Classification of Diseases, 10th Edition (ICD-10) for cancer specific mortality, while for cardiovascular (CV) mortality, we included diseases of the heart and circulation system (ICD-10 = I00–I178).

### Measurement of PLR and definition of the MAMC tertiles group

Platelet-to-lymphocyte ratio (PLR) is the ratio of the absolute platelet count to the absolute lymphocyte count. In NHANES III, complete blood count (CBC) was performed automatically using the Coulter method with different generations of Coulter counters. In the present study, PLR cut-off point was the median PLR of the participants.

The data of MAC was collected according to the standard procedures defined by Lohman and colleagues and was based on the age of the participant [9]. They were divided into tertiles and the lowest tertile was used as the reference group. All participants were divided into low PLR and high PLR groups with the cut-off point being the median PLR level, and each group was divided into three subgroups (lowest, middle, and highest) based on their MAMC level. The tertiles were as follows: T1 (15.3–24.1 cm), T2 (24.2–28.1 cm), and T3 (28.2–44.1 cm) cm in the low PLR group, and T1 (13.8–23.3 cm), T2 (23.4–27.3 cm), and T3 (27.4–41.1 cm) in the high PLR group.

### Covariates

The participants were interviewed for self-reporting of general characteristics and previous medical histories regarding the following variables: age, race/ethnicity, sex, smoking status, and physician-diagnosed previous clinical illnesses (congestive heart failure–CHF, stroke, asthma, malignancy, and type 2 diabetes mellitus–DM). Diabetes was defined as self-report history, fasting plasma glucose ≥ 126 mg/dL, OGTT 2-h plasma glucose ≥ 200 mg/dL, or random serum glucose ≥ 200 mg/dL. The biochemistry profiles were collected from the blood samples, which were then assessed by the Lipoprotein Analytical Laboratory at Johns Hopkins University, Baltimore, Maryland, USA. The plasma glucose level was assessed via the hexokinase enzymatic method. Serum total cholesterol (TC), triglycerides (TG), high-density lipoprotein (HDL), and low-density lipoprotein (LDL) levels were measured using the Hitachi 704 Analyzer, and serum C-reactive protein (CRP) levels were measured using latex-enhanced nephelometry. Other biochemical profiles, such as serum albumin, aspartate transaminase (AST), alanine transaminase (ALT), uric acid (UA), and total bilirubin were measured by using Beckman Synchron LX20.

### Statistical analysis

The statistical analyses were performed using SPSS version 18 (SPSS, Inc., Chicago, IL, USA). Continuous data were compared using the Independent t-test or Wilcoxon Rank sum test, and presented as means ± standard errors (SE). Categorical data were compared using the Chi-square test and presented as number and percentages (%). The p values of less than 0.05 were considered to be statistically significant.

The association between MAMC tertiles and all-cause, cardiovascular, and cancer mortalities were evaluated by Cox proportional hazard models. An extended-model approach was used for adjusted covariates. Model 1 was not adjusted for any variables; model 2 was adjusted for race, sex, and body mass index (BMI); model 3 was adjusted for the variables in model 2 and systolic blood pressure, serum triglycerides, and serum fasting glucose; and model 4 was adjusted for the variables of models 2 and 3, and smoking, congestive heart failure, stroke, gout, and malignancy.

### Ethics statement

The National Center for Health Statistics Institutional Review Board approved the NHANES III study, and all participants in the NHANES III provided written informed consent. This study was exempt from IRB review because we analyzed an open available online database. All methods in this study were performed based on the relevant guidelines and regulations.

## Results

The study included 14,221 adults with 6,701 (47.1%) male and 7,520 (52.9%) female participants. The median PLR ratio in this population was 122. The participants were divided into two groups; low PLR group (PLR<122) and high PLR (PLR≥122) group. The mean MAMC was 26.3 ± 3.9 cm and mean age was 47.5 ± 18.7 years in the lower PLR group. The mean MAMC was 25.6 ± 3.8 cm and the mean age was 47.8 ± 19.4 years in the high PLR group. In our study, the mean follow-up duration was 14.3 years.

The clinical characteristics of the participants of the low PLR group related to mortality are depicted in Table 1. In this group, higher levels of age, systolic blood pressure (SBP), diastolic blood pressure (DBP), and serum levels of TG, TC, LDL, CRP, total bilirubin, UA, glucose, and AST were associated with increased risk of mortality. Other factors that were associated with increased risk of mortality included male sex, non-Hispanic white ethnicity, and associated conditions like congestive heart failure, stroke, asthma, malignancy, diabetes mellitus, and smoking. However, higher serum levels of HDL, albumin, and ALT were associated with decreased risk of mortality.

**Table 1:** Characteristics of participants with low platelet-to-lymphocyte ratio (<122).

Characteristics of Study Participants	Non-mortality	Mortality	Total	P value
	n = 5,447	n = 1663	n = 7110	
**Continuous variables, mean (SE)**				
MAMC (cm)	26.2 (4.0)	26.3 (3.6)	26.3 (3.9)	0.630
Age (years)	41.4 (15.2)	67.4 (14.9)	47.5 (18.7)	<0.001
BMI (kg/m^2^)	27.6 (5.9)	27.5 (5.5)	27.5 (5.8)	0.577
SBP (mmHg)	121.2 (18.4)	141.3 (25.0)	125.9 (21.8)	<0.001
DBP (mmHg)	72.4 (12.7)	73.8 (14.2)	72.7 (13.1)	<0.001
Serum TG (mg/dL)	148.3 (115.1)	174.5 (125.5)	154.5 (118.1)	<0.001
Serum total Cholesterol (mg/dL)	202.3 (43.6)	217.4 (48.5)	205.9 (45.2)	<0.001
LDL cholesterol (mg/dL)	126.1 (38.4)	136.9 (40.7)	128.6 (39.2)	<0.001
HDL cholesterol (mg/dL)	49.9 (15.1)	48.5 (16.2)	49.6 (15.3)	0.001
Serum CRP (mg/dL)	0.4 (0.6)	0.5 (0.6)	0.4 (0.6)	<0.001
Serum total bilirubin (mg/dL)	0.6 (0.3)	0.6 (0.4)	0.6 (0.4)	<0.001
Serum UA (mg/dL)	5.3 (1.4)	5.9 (1.6)	5.5 (1.5)	<0.001
Serum glucose (mg/dL)	97.2 (31.6)	116.1 (54.1)	101.6 (38.9)	<0.001
Albumin (g/dL)	4.2 (0.4)	4.0 (0.4)	4.1 (0.4)	<0.001
AST (U/L)	22.9 (16.0)	25.3 (24.5)	23.5 (18.4)	<0.001
ALT (U/L)	20.2 (19.6)	17.2 (18.7)	19.5 (19.4)	<0.001
**Categorical variables, N (%)**				
Male	2604 (47.8)	966 (58.1)	3570 (50.2)	<0.001
Non-Hispanic white	1933 (35.5)	896 (53.9)	2829 (39.8)	<0.001
Congestive heart failure	82 (1.5)	170 (10.2)	252 (3.5)	<0.001
Stroke	57 (1)	135 (8.1)	192 (2.7)	<0.001
Asthma	340 (6.2)	124 (7.5)	464 (6.5)	0.041
Malignancy	118 (2.2)	116 (7.0)	234 (3.3)	<0.001
Type 2 diabetes mellitus	302 (5.5)	341 (20.5)	643 (9.0)	<0.001
Smoking	488 (9.0)	326 (19.6)	814 (11.4)	<0.001

Abbreviation: N, number; SD, standard deviation; MAMC, mid-arm muscle circumference; BMI, body mass index; SBP, systolic blood pressure; DBP, diastolic blood pressure; Serum TG, serum total triglycerides; HDL, high-density lipoprotein; LDL, low-density lipoprotein; Serum CRP, serum C-reactive protein; Serum UA, serum uric acid; AST, aspartate aminotransferase; ALT, alanine aminotransferase.

The clinical characteristics of the participants of the high PLR group related to mortality are depicted in Table 2. In this group, higher levels of age, SBP, and serum TG, TC, LDL, CRP, UA, and glucose were associated with increased risk of mortality. Additional factors that increased the risk of mortality included male sex, non-Hispanic white ethnicity, and conditions such as congestive heart failure, stroke, asthma, malignancy, diabetes mellitus, and smoking. However, high levels of BMI, and serum albumin and ALT were associated with decreased risk of mortality.

**Table 2:** Characteristics of participants with high platelet-to-lymphocyte ratio (≥122).

Characteristics of Study Participants	Non-mortality	Mortality	Total	P value
	n = 5389	n = 1722	n = 7111	
**Continuous variables, mean (SE)**				
MAMC (cm)	25.6 (3.9)	25.5 (3.5)	25.6 (3.8)	0.443
Age (years)	41.1 (15.3)	69.0 (15.1)	47.8 (19.4)	<0.001
BMI (kg/m^2^)	26.9 (5.6)	26.5 (5.4)	26.8 (5.6)	0.007
SBP (mmHg)	119.9 (18.2)	141.8 (25.0)	125.2 (22.1)	<0.001
DBP (mmHg)	72.1 (12.4)	72.7 (15.5)	72.3 (13.2)	0.145
Serum TG (mg/dL)	128.6 (102.0)	154.5 (142.3)	134.9 (113.6)	<0.001
Serum total Cholesterol (mg/dL)	202.0 (42.9)	218.4 (47.0)	206.0 (44.5)	<0.001
LDL cholesterol (mg/dL)	124.7 (36.7)	137.2 (42.5)	127.4 (39.0)	<0.001
HDL cholesterol (mg/dL)	52.6 (15.0)	52.6 (17.7)	52.6 (15.7)	0.976
Serum CRP (mg/dL)	0.5 (0.8)	0.7 (1.3)	0.5 (0.9)	<0.001
Serum total bilirubin (mg/dL)	0.6 (0.3)	0.6 (0.3)	0.6 (0.3)	0.847
Serum UA (mg/dL)	5.1 (1.4)	5.7 (1.6)	5.3 (1.5)	<0.001
Serum glucose (mg/dL)	95.1 (27.3)	111.4 (51.9)	99.1 (35.6)	<0.001
Albumin (g/dL)	4.2 (0.4)	4.0 (0.4)	4.1 (0.4)	<0.001
AST (U/L)	21.3 (12.7)	21.6 (14.4)	21.4 (13.2)	0.375
ALT (U/L)	17.6 (14.3)	13.9 (14.2)	16.7 (14.4)	<0.001
**Categorical variables, N (%)**				
Male	2103 (39)	1028 (59.7)	3131 (44)	<0.001
Non-Hispanic white	1933 (35.5)	896 (53.9)	2829 (39.8)	<0.001
Congestive heart failure	78 (1.4)	158 (9.2)	236 (3.3)	<0.001
Stroke	37 (0.7)	134 (7.8)	171 (2.4)	<0.001
Asthma	377 (7.0)	145 (8.4)	522 (7.3)	0.048
Malignancy	106 (2.0)	190 (11.0)	296 (4.2)	<0.001
Type 2 diabetes mellitus	223 (4.1)	258 (15.0)	481 (6.8)	<0.001
Smoking	458 (8.5)	303 (17.6)	761 (10.7)	<0.001

Abbreviation: N, number; SD, standard deviation; MAMC, mid-arm muscle circumference; BMI, body mass index; SBP, systolic blood pressure; DBP, diastolic blood pressure; Serum TG, serum total triglycerides; HDL, high-density lipoprotein; LDL, low-density lipoprotein; Serum CRP, serum C-reactive protein; Serum UA, serum uric acid; AST, aspartate aminotransferase; ALT, alanine aminotransferase.

Table 3 presents the clinical characteristics of the participants of the low PLR group related to MAMC tertiles. In these participants, higher levels of BMI, SBP, DBP, TC, TG, LDL, total bilirubin, UA, glucose, albumin, AST, and ALT were associated with higher MAMC tertiles. However, higher levels of HDL were associated with lower MAMC tertiles. Higher percentages of male participants and those with diabetes mellitus and smoking were associated with higher MAMC tertiles. Higher percentages of non-Hispanic white participants were associated with lower MAMC tertiles. Higher levels of age and higher percentages of congestive heart failure and malignancy were found in the second tertile of MAMC.

**Table 3:** Characteristics of participants with low platelet-to-lymphocyte ratio (<122) according to mid-arm muscle circumference tertiles.

Characteristics of the study participants	Tertiles of mid-arm muscle circumference (cm)			Total	P for trend
	T1 (15.3–24.1 cm)	T2 (24.2–28.1 cm)	T3 (28.2–44.1 cm)		
**Continuous variables, mean (SE)**					
MAMC (cm)	21.7 (1.4)	25.8 (1.2)	30.4 (2.1)	26.3 (3.9)	<0.001
Age (years)	45.4 (19.7)	50.5 (19.8)	46.6 (16.7)	47.5 (18.8)	<0.001
BMI (kg/m^2^)	24.1 (4.1)	27.3 (4.9)	29.5 (5.3)	27.1 (5.3)	<0.001
SBP (mmHg)	120.1 (23.1)	127.5 (22.2)	128.5 (19.2)	125.6 (21.8)	<0.001
DBP (mmHg)	68.1 (13.0)	72.1 (12.9)	76.8 (11.7)	72.7 (13.0)	<0.001
Serum TG (mg/dL)	128.9 (101.6)	153.7 (110.0)	175.4 (132.9)	154.3 (118.2)	<0.001
Serum total cholesterol (mg/dL)	203.4 (47.8)	206.7 (45.3)	206.8 (43.2)	205.7 (45.3)	0.017
LDL-cholesterol (mg/dL)	122.0 (39.4)	129.3 (39.9)	132.3 (37.9)	128.4 (39.2)	<0.001
HDL-cholesterol (mg/dL)	56.1 (16.3)	49.5 (14.8)	44.6 (13.2)	49.7 (15.4)	<0.001
Serum CRP (mg/dL)	0.4 (0.5)	0.4 (0.6)	0.4 (0.6)	0.4 (0.6)	0.100
Serum total bilirubin (mg/dL)	0.5 (0.3)	0.6 (0.3)	0.7 (0.4)	0.6 (0.4)	<0.001
Serum UA (mg/dL)	4.6 (1.3)	5.5 (1.4)	6.1 (1.4)	5.4 (1.5)	<0.001
Serum glucose (mg/dL)	95.5 (35.6)	102.9 (38.8)	104.7 (39.4)	101.3 (38.3)	<0.001
Albumin (g/dL)	4.1 (0.4)	4.1 (0.4)	4.2 (0.4)	4.1 (0.4)	<0.001
AST (U/L)	20.8 (14.4)	23.7 (20.4)	25.5 (18.8)	23.5 (18.3)	<0.001
ALT (U/L)	15.3 (16.1)	18.6 (17.2)	23.4 (22.2)	19.4 (19.2)	<0.001
**Categorical variables, N (%)**					
Male	120 (5.8)	1158 (50.9)	2228 (87.7)	3506 (50.9)	<0.001
Non-Hispanic white	938 (45.3)	927 (40.7)	896 (35.3)	2761 (40.1)	<0.001
Congestive heart failure	46 (2.2)	99 (4.4)	97 (3.8)	242 (3.5)	0.007
Stroke	47 (2.3)	76 (3.3)	58 (2.3)	181 (2.6)	0.085
Asthma	125 (6.0)	144 (6.3)	177 (7.0)	446 (6.5)	0.481
Malignancy	70 (3.4)	92 (4.0)	64 (2.5)	226 (3.3)	0.027
Type 2 diabetes mellitus	129 (6.2)	222 (9.8)	258 (10.2)	609 (8.8)	<0.001
Smoking	33 (1.6)	255 (11.2)	514 (20.2)	802 (11.6)	<0.001

Abbreviation: N, number; SEs, standard errors; MAMC, mid-arm muscle circumference; BMI, body mass index; SBP, systolic blood pressure; DBP, diastolic blood pressure; Serum TG, serum total triglycerides; HDL, high-density lipoprotein; LDL, low-density lipoprotein; Serum CRP, serum C-reactive protein; Serum UA, serum uric acid; AST, aspartate aminotransferase; ALT, alanine aminotransferase.

Table 4 shows the clinical characteristics of the participants of the higher PLR group related to MAMC tertiles. In these participants, higher levels of BMI, DBP, and serum TG, LDL, total bilirubin, UA, glucose, albumin, AST, and ALT were associated with higher MAMC tertiles. However, higher HDL was associated with lower MAMC tertiles. Higher levels of age, SBP, and serum TC and CRP were found in the second tertile of MAMC. Higher percentages of male participants and those with smoking habit were associated with higher MAMC tertiles. Higher percentages of non-Hispanic white participants and those with malignancies were associated with lower MAMC tertiles. The maximal percentage of patients with congestive heart failure, stroke, and diabetes mellitus were found in the second tertile of MAMC.

**Table 4:** Characteristics of participants with high platelets-to-lymphocytes ratio (≥122) according to mid-arm muscle circumference tertiles.

Characteristics of the study participants	Tertiles of mid-arm muscle circumference (cm)			Total	P for trend
	T1 (13.8–23.3 cm)	T2 (23.4–27.3 cm)	T3 (27.4–41.1 cm)		
**Continuous variables, mean (SE)**					
MAMC (cm)	21.7 (1.4)	25.7 (1.2)	30.3 (2.1)	25.6 (3.8)	<0.001
Age (years)	46.7 (20.2)	51.1 (20.1)	46.0 (17.0)	48.0 (19.4)	<0.001
BMI (kg/m^2^)	23.9 (4.0)	27.0 (4.8)	29.0 (5.2)	26.5 (5.1)	<0.001
SBP (mmHg)	120.3 (23.2)	128.1 (23.2)	127.5 (18.5)	125.1 (22.2)	<0.001
DBP (mmHg)	68.1 (13.185)	72.5 (12.6)	76.8 (12.3)	72.2 (13.2)	<0.001
Serum TG (mg/dL)	116.2 (91.4)	135.8 (102.6)	156.2 (141.9)	134.7 (113.4)	<0.001
Serum total cholesterol (mg/dL)	202.9 (44.5)	207.9 (46.3)	207.8 (42.5)	206.1 (44.6)	<0.001
LDL cholesterol (mg/dL)	122.0 (38.4)	129.2 (38.2)	131.8 (37.9)	127.3 (38.4)	<0.001
HDL cholesterol (mg/dL)	57.5 (15.5)	52.0 (15.9)	47.6 (14.2)	52.7 (15.8)	<0.001
Serum CRP (mg/dL)	0.5 (1.0)	0.6 (0.9)	0.5 (0.8)	0.5 (0.9)	<0.001
Serum total bilirubin (mg/dL)	0.5 (0.3)	0.6 (0.3)	0.7 (0.3)	0.6 (0.3)	<0.001
Serum UA (mg/dL)	4.5 (1.2)	5.3 (1.4)	6.1 (1.4)	5.3 (1.5)	<0.001
Serum glucose (mg/dL)	93.7 (31.3)	101.3 (37.1)	102.9 (37.9)	99.0 (35.6)	<0.001
Albumin (g/dL)	4.1 (0.4)	4.1 (0.4)	4.2 (0.4)	4.1 (0.4)	<0.001
AST (U/L)	19.4 (10.1)	21.4 (15.3)	23.8 (13.6)	21.4 (13.2)	<0.001
ALT (U/L)	13.4 (9.8)	16.0 (14.1)	21.5 (17.4)	16.7 (14.2)	<0.001
**Categorical variables, N (%)**					
Male	150 (5.9)	1133 (48.2)	1819 (87.8)	3102 (44.6)	<0.001
Non-Hispanic white	1227 (48.3)	1037 (44.1)	833 (40.2)	3097 (44.5)	<0.001
Congestive heart failure	60 (2.4)	106 (4.5)	67 (3.2)	233 (3.3)	0.001
Stroke	50 (2.0)	79 (3.4)	39 (1.9)	168 (2.4)	0.005
Asthma	167 (6.6)	177 (7.5)	162 (7.8)	506 (7.3)	0.226
Malignancy	132 (5.2)	106 (4.5)	52 (2.5)	290 (4.2)	<0.001
Type 2 diabetes mellitus	118 (4.6)	201 (8.5)	145 (7.0)	464 (6.7)	<0.001
Smoking	43 (1.7)	257 (10.9)	456 (22)	756 (10.9)	<0.001

Abbreviation: N, number; SEs, standard errors; MAMC, mid-arm muscle circumference; BMI, body mass index; SBP, systolic blood pressure; DBP, diastolic blood pressure; Serum TG, serum total triglycerides; HDL, high-density lipoprotein; LDL, low-density lipoprotein; Serum CRP, serum C-reactive protein; Serum UA, serum uric acid; AST, aspartate aminotransferase; ALT, alanine aminotransferase.

We further investigated the relationship between MAMC and all-cause mortality, cancer mortality, and CV mortality according to the low and high PLR values. The results of multivariable adjusted analyses for all-cause mortality, cancer mortality, and CV mortality associated with MAMC in the two PLR groups are shown in Table 5. After adjusting for all the covariates (model 4), in the high PLR group, the highest MAMC tertile was significantly associated with decreasing hazards ratios (HRs) for all-cause and cancer mortalities compared with the lowest MAMC tertile. However, this association was not observed in the low PLR group. Furthermore, higher MAMC tertiles were not significantly associated with decreasing CV mortality after adjusting for covariates in both the groups.

**Table 5:** Cox proportional hazards ratios of all-cause, cardiovascular, and cancer mortalities according to mid-arm muscle circumference in American individuals.

PLR (<122)				PLR (≥122)			
Models ^a^	Tertiles of MAMC	Hazard Ratio (95%CI)	*P* -value	Models ^a^	Tertiles of MAMC	Hazard Ratio (95%CI)	*P* -value
**All-cause mortality**							
Model 1	T2 vs. T1	1.83 (1.57–2.12)	<0.001	Model 1	T2 vs. T1	1.51 (1.32–1.73)	<0.001
	T3 vs. T1	1.20 (1.02–1.40)	0.026		T3 vs. T1	0.94 (0.81–1.10)	0.453
Model 2	T2 vs. T1	1.11 (0.94–1.31)	0.211	Model 2	T2 vs. T1	0.97 (0.84–1.13)	0.734
	T3 vs. T1	0.95 (0.78–1.15)	0.608		T3 vs. T1	0.82 (0.67–0.99)	0.041
Model 3	T2 vs. T1	1.09 (0.92–1.28)	0.326	Model 3	T2 vs. T1	0.95 (0.81–1.10)	0.472
	T3 vs. T1	0.90 (0.74–1.09)	0.273		T3 vs. T1	0.79 (0.65–0.96)	0.016
Model 4	T2 vs. T1	1.09 (0.92–1.28)	0.337	Model 4	T2 vs. T1	0.91 (0.78–1.06)	0.244
	T3 vs. T1	0.89 (0.73–1.08)	0.238		T3 vs. T1	0.76 (0.62–0.92)	0.006
**Cancer mortality**							
Model 1	T2 vs. T1	1.77 (1.31–2.40)	<0.001	Model 1	T2 vs. T1	2.04 (1.53–2.71)	<0.001
	T3 vs. T1	1.20 (0.88–1.64)	0.254		T3 vs. T1	1.30 (0.95–1.78)	0.097
Model 2	T2 vs. T1	1.05 (0.75–1.47)	0.769	Model 2	T2 vs. T1	0.96 (0.70–1.33)	0.803
	T3 vs. T1	0.86 (0.58–1.28)	0.463		T3 vs. T1	0.61 (0.41–0.90)	0.013
Model 3	T2 vs. T1	1.06 (0.76–1.47)	0.748	Model 3	T2 vs. T1	0.91 (0.66–1.27)	0.585
	T3 vs. T1	0.87 (0.58–1.29)	0.486		T3 vs. T1	0.58 (0.39–0.86)	0.007
Model 4	T2 vs. T1	1.06 (0.76–1.48)	0.743	Model 4	T2 vs. T1	0.88 (0.63–1.22)	0.473
	T3 vs. T1	0.86 (0.57–1.28)	0.444		T3 vs. T1	0.58 (0.39–0.86)	0.008
**CV mortality**							
Model 1	T2 vs. T1	2.00 (1.59–2.51)	<0.001	Model 1	T2 vs. T1	1.58 (1.29–1.93)	<0.001
	T3 vs. T1	1.17 (0.92–1.49)	0.201		T3 vs. T1	0.96 (0.76–1.21)	0.719
Model 2	T2 vs. T1	1.08 (0.84–1.38)	0.563	Model 2	T2 vs. T1	1.08 (0.87–1.35)	0.486
	T3 vs. T1	0.94 (0.70–1.25)	0.657		T3 vs. T1	1.07 (0.80–1.42)	0.656
Model 3	T2 vs. T1	1.05 (0.82–1.34)	0.716	Model 3	T2 vs. T1	1.04 (0.84–1.30)	0.711
	T3 vs. T1	0.87 (0.65–1.17)	0.356		T3 vs. T1	1.03 (0.78–1.38)	0.817
Model 4	T2 vs. T1	1.06 (0.83–1.36)	0.645	Model 4	T2 vs. T1	0.98 (0.78–1.22)	0.845
	T3 vs. T1	0.87 (0.64–1.17)	0.339		T3 vs. T1	0.95 (0.71–1.27)	0.728

^a^ Adjusted covariates

Model 1 = Unadjusted.

Model 2 = adjusted for sex, race, and body mass index.

Model 3 = Model 2 + adjusted for systolic blood pressure, serum triglycerides, and serum fasting glucose.

Model 4 = Model 3 + adjusted for history of smoking, congestive heart failure, stroke, gout, and malignancy.

## Discussion

PLR is an inexpensive and routinely available valuable maker for nonspecific inflammatory conditions [10]. Previous reports have shown that inflammation plays a critical role in cancer progression [11],coronary artery disease [12], chronic kidney disease [13], heart failure [14], and all-cause mortality [15]. Recently, studies demonstrated that PLR is an inflammatory biomarker, prognostic factor, and mortality predictor in patients with malignancy, cardiovascular diseases, and end-stage renal diseases [1–4]. The mechanisms of the influence of PLR on prognoses in patients with malignancies, cardiovascular diseases, and end-stage renal disease is still incompletely understood. The increased inflammatory response may promote production of acute phase proteins and cytokines, and lead to the differentiation of megakaryocytes into platelets [16–18]. Platelets play a critical role in thrombosis and inflammation. The increased platelet counts may lead to the release of pro-inflammatory cytokines and further result in a pro-thrombotic state, which will lead to further platelet activation and thrombosis [19–20]. Platelets also play a critical role in cancer cell proliferation and migration through production of metalloproteinases and growth factors [21–22]. Lymphocytes also play a major role in inflammation; under stressful conditions, the activation of hypothalamic-pituitary-adrenal axis results in secretion of cortisol, which results in decreased lymphocyte count [23–24].

Since the relationships between high PLR, mortality, and clinical outcomes in several diseases have been proven, we should attempt to identify the potential factors to decrease the mortality in individuals with high PLR. Patients with lower MAMC may have reduced lean muscle mass and decreased muscle strength, which may be due to decreased physical activity or an underlying medical disease [7, 25]. In previous studies, MAMC has been demonstrated to be associated with physical function, life quality [7], male all-cause mortality [26], and mortality in patients on hemodialysis [5].

In our study, we examined a nationally demonstrative sample of American adults to determine whether MAMC influences mortality outcomes in low and high PLR groups. We observed a statistically significant decrease in the HRs for all-cause and cancer mortalities in the highest MAMC tertile compared with those in the lowest MAMC tertile after adjusting for covariates; however, this association was not observed in the low PLR group. Individuals with high PLR are believed to be in a state of systemic inflammation and one of the reasons for our results is that higher MAMC may have a protective effect from mortality in such individuals. However, patients with lower MAMC may be suffering from weakness, chronic illness, or malnutrition; therefore, the underlying condition may result in a higher mortality rate in individuals with chronic inflammation. Previous studies reported that physical performance was associated with MAMC [27] and physical activity may aid in the prevention and treatment of sarcopenic obesity [28]. As mentioned above, proper exercise may help control inflammation, and it plays an important role in lowering the mortality risk in individuals with high PLR. Routine exercise maintains muscle mass and strength, and it also improves inflammatory conditions by reducing the levels of pro-inflammatory cytokines [29–31]. Therefore, we may suppose that proper exercising modulates inflammation and leads to decreased mortality risk in the population with high PLR.

There are several limitations to our study. First, the cross-sectional nature of our survey and the fact that the anthropometric and blood examination data were collected only at the follow-up time may have resulted in bias. Second, the large amount of data was collected from self-reporting and, therefore, maybe have a recall bias. Third, even after adjusting for many confounding variables, some confounding bias may still be present. Fourth, the native design of NHANES database is not fit for a global population.

## Conclusion

In conclusion, the highest MAMC tertile was significantly associated with decreasing HRs for all-cause and cancer mortalities compared with the lowest MAMC tertile in individuals with PLR ≥ 122. Clinical care should focus on maintaining muscle mass and muscle strength by means of increasing physical activity and controlling the underlying chronic inflammatory disease to decrease the mortality rate. Future studies should focus on the longitudinal investigation of the changes in MAMC and the change in the influence of MAMC on mortality in individuals with high PLR.

## References

[1] Templeton AJ, Ace O, McNamara MG. Prognostic role of platelet to lymphocyte ratio in solid tumors: a systematic review and meta-analysis. Cancer Epidemiol Biomarkers Prev 2014;23(7):1204–12. 10.1158/1055-9965.EPI-14-0146 24793958 10.1158/1055-9965.EPI-14-0146

[2] You J, Zhu GQ, Xie L. Preoperative platelet to lymphocyte ratio is a valuable prognostic biomarker in patients with colorectal cancer. Oncotarget 2016;7(18):25516–27. doi: 10.18632/oncotarget.8334 27027440 10.18632/oncotarget.8334 PMC5041922

[3] Yaprak M, Turan MN, Dayanan R, Akın S, Değirmen E, Yıldırım M, et al Platelet-to-lymphocyte ratio predicts mortality better than neutrophil-to-lymphocyte ratio in hemodialysis patients. Int Urol Nephrol 2016;48(8):1343–48. 10.1007/s11255-016-1301-4 27118565 10.1007/s11255-016-1301-4

[4] Akkaya E, Gul M, Ugur M. Platelet to lymphocyte ratio: a simple and valuable prognostic marker for acute coronary syndrome. Int J Cardiol 2014;177(2):597–8. 10.1016/j.ijcard.2014.08.143 25220175 10.1016/j.ijcard.2014.08.143

[5] Noori N, Kopple JD, Kovesdy CP, Feroze U, Sim JJ, Murali SB, et al Mid-arm muscle circumference and quality of life and survival in maintenance hemodialysis patients. Clin J Am Soc Nephrol 2010;5(12):2258–68. 10.2215/CJN.02080310 20947789 10.2215/CJN.02080310 PMC2994088

[6] Atlantis E, Martin SA, Haren MT, Taylor AW, Wittert GA; Members of the Florey Adelaide Male Ageing Study. Inverse associations between muscle mass, strength, and the metabolic syndrome. Metabolism 2009;58(7):1013–22. 10.1016/j.metabol.2009.02.027 19394973 10.1016/j.metabol.2009.02.027

[7] Landi F, Russo A, Liperoti R, Pahor M, Tosato M, Capoluongo E, et al Midarm muscle circumference, physical performance and mortality: results from the aging and longevity study in the Sirente geographic area ilSIRENTE study. Clin Nutr 2009;29(4):441–8. 10.1016/j.clnu.2009.12.006 20116909 10.1016/j.clnu.2009.12.006

[8] Hyattsville, National Center for Health Statistics. Plan and operation of the Third National Health and Nutrition Examination Survey, 1988–94. Series 1: programs and collection procedures. Vital Health Stat 1 1994; (32):1–407. 7975354

[9] Lohman TG, Roche AF, Martorell R, eds. Anthropometric Standardization Reference Manual. Human Kinetics Books, 1988.

[10] Balta S, Demırkol S, Kucuk U. The platelet lymphocyte ratio may be useful inflammatory indicator in clinical practice. Hemodial Int 2013;17(4):668–9. 10.1111/hdi.12058 23763539 10.1111/hdi.12058

[11] Mantovani A, Allavena P, Sica A, Balkwill F. Cancer-related inflammation. Nature 2008;454(7203):436–44. 10.1038/nature07205 18650914 10.1038/nature07205

[12] Kruk M, Przyłuski J, Kalińczuk Ł, Pregowski J, Deptuch T, Kadziela J, et al Association of non-specific inflammatory activation with early mortality in patients with ST-elevation acute coronary syndrome treated with primary angioplasty. Circ J 2008;72(2):205–11. 18219155 10.1253/circj.72.205

[13] Okyay GU, Inal S, Oneç K, Er RE, Paşaoğlu O, Paşaoğlu H, et al Neutrophil to lymphocyte ratio in evaluation of inflammation in patients with chronic kidney disease. Ren Fail 2013;35(1):29–36. 10.3109/0886022X.2012.734429 23113674 10.3109/0886022X.2012.734429

[14] Yndestad A. et al Systemic inflammation in heart failure: the whys and wherefores. Heart Fail Rev 2006;11(1):83–92. 10.1007/s10741-006-9196-2 16819581 10.1007/s10741-006-9196-2

[15] Zuo H, Ueland PM, Ulvik A, Eussen SJ, Vollset SE, Nygård O, et al Plasma Biomarkers of Inflammation, the Kynurenine Pathway, and Risks of All-Cause, Cancer, and Cardiovascular Disease Mortality: The Hordaland Health Study. Am J Epidemiol 2016;183(4):249–258. 10.1093/aje/kwv242 26823439 10.1093/aje/kwv242 PMC4753283

[16] Imai T, Koike K, Kubo T, Kikuchi T, Amano Y, Takagi M, et al Interleukin-6 supports human megakaryocytic proliferation and differentiation in vitro. Blood 1991;78(8):1969–74. 1912578

[17] Waehre T, Damås JK, Yndestad A, Taskén K, Pedersen TM, Smith C, et al Effect of activated platelets on expression of cytokines in peripheral blood mononuclear cells—potential role of prostaglandin E2. Thromb Haemost 2004;92(6):1358–67. 10.1160/TH04-03-0146 15583745 10.1160/TH04-03-0146

[18] Damås JK, Waehre T, Yndestad A, Otterdal K, Hognestad A, Solum NO, et al Interleukin-7-mediated inflammation in unstable angina: possible role of chemokines and platelets. Circulation 2003;107(21):2670–6. 10.1161/01.CIR.0000070542.18001.87 12742982 10.1161/01.CIR.0000070542.18001.87

[19] Hansson GK. Inflammation atherosclerosis and coronary artery disease. N Engl J Med 2005;352(16):1685–95. 10.1056/NEJMra043430 15843671 10.1056/NEJMra043430

[20] Ferroni P, Basili S, Davi G. Platelet activation, inflammatory mediators and hypercholesterolemia. Curr Vasc Pharmacol 2003;1(2):157–69. 15320841 10.2174/1570161033476772

[21] Egan K, Crowley D, Smyth P, O'Toole S, Spillane C, Martin C, et al Platelet adhesion and degranulation induce pro-survival and pro-angiogenic signalling in ovarian cancer cells. PLoS One 2011;6(10):26125 10.1371/journal.pone.0026125 22022533 10.1371/journal.pone.0026125 PMC3192146

[22] Suzuki K, Aiura K, Ueda M, Kitajima M. The influence of platelets on the promotion of invasion by tumor cells and inhibition by antiplatelet agents. Pancreas 2004;29(2):132–40. 15257105 10.1097/00006676-200408000-00008

[23] Maisel AS, Knowlton KU, Fowler P, Rearden A, Ziegler MG, Motulsky HJ, et al Adrenergic control of circulating lymphocyte subpopulations: effects of congestive heart failure, dynamic exercise, and terbutaline treatment. J Clin Invest 1990;85(2):462–7. 10.1172/JCI114460 2153706 10.1172/JCI114460 PMC296446

[24] Ommen SR, Hodge DO, Rodeheffer RJ, McGregor CG, Thomson SP, Gibbons RJ. Predictive power of the relative lymphocyte count in patients with advanced heart failure. Circulation. 1998;97(1):19–22. 9443426 10.1161/01.cir.97.1.19

[25] Roubenoff R. Sarcopenia and its implications for the elderly. Eur J Clin Nutrition. 2000;54 Supplement 3:S40–7. 11041074 10.1038/sj.ejcn.1601024

[26] Wu LW, Lin YY, Kao TW, Lin CM, Liaw FY, Wang CC, et al Mid-arm muscle circumference as a significant predictor of all-cause mortality in male individuals. PLoS One 2017;12(2):e0171707 10.1371/journal.pone.0171707 28196081 10.1371/journal.pone.0171707 PMC5308605

[27] Landi F, Russo A, Liperoti R, Pahor M, Tosato M, Capoluongo E, et al Midarm muscle circumference, physical performance and mortality: Results from the aging and longevity study in the Sirente geographic area (ilSIRENTE study). Clin Nutr 2010;29(4):441–7. 10.1016/j.clnu.2009.12.006 20116909 10.1016/j.clnu.2009.12.006

[28] Lee DC, Shook RP, Drenowatz C, Blair SN. Physical activity and sarcopenic obesity: definition, assessment, prevalence and mechanism. Future Sci OA. 2016;2(3):FSO127 10.4155/fsoa-2016-0028 28031974 10.4155/fsoa-2016-0028 PMC5137918

[29] Adamopoulos S, Parissis J, Kroupis C, Georgiadis M, Karatzas D, Karavolias G, et al Physical training reduces peripheral markers of inflammation in patients with chronic heart failure. Eur Heart J 2001;22(9):791–7. 10.1053/euhj.2000.2285 11350112 10.1053/euhj.2000.2285

[30] Hur S, Han GS, Cho BJ. Changes in Glucose, TNF-α and IL-6 Blood Levels in Middle-aged Women Associated with Aerobic Exercise and Meditation Training. J Phys Ther Sci 2014;26(12):1933–6. 10.1589/jpts.26.1933 25540501 10.1589/jpts.26.1933 PMC4273061

[31] Nishida Y, Tanaka K, Hara M, Hirao N, Tanaka H, Tobina T, et al Effects of home-based bench step exercise on inflammatory cytokines and lipid profiles in elderly Japanese females: A randomized controlled trial. Arch Gerontol Geriatr 2015;61(3):443–51. 10.1016/j.archger.2015.06.017 26228714 10.1016/j.archger.2015.06.017

